# Efficacy of Hypertension Self-Management Classes Among Patients at a Federally Qualified Health Center

**DOI:** 10.5888/pcd18.200628

**Published:** 2021-07-15

**Authors:** Cameron Eck, Holly Biola, Tiffany Hayes, Dominique Bulgin, Colette Whitney, Rohith Raman, Melanie Bakovic, Awanya Caesar, Rosa Becerra-Soberon, Joan Chaplin, Bradi B. Granger

**Affiliations:** 1 Duke-Margolis Center for Health Policy, Durham, North Carolina; 2 Lincoln Community Health Center, Durham, North Carolina; 3 Duke University School of Nursing, Durham, North Carolina; 4 Duke University School of Medicine, Durham, North Carolina; 5 Duke Clinical and Translational Science Institute, Durham, North Carolina; 6 University of Tennessee, Knoxville College of Nursing, Knoxville, Tennessee

## Abstract

Structural racism has contributed to persistent racial disparities in hypertension control, with Black men suffering the highest prevalence of uncontrolled hypertension. Lincoln Community Health Center, our urban Federally Qualified Health Center (FQHC), aimed to use hypertension self-management classes to improve hypertension control among our clinic patients, particularly Black men. Patients attending classes learned about hypertension, were given blood pressure cuffs to use at home, and had the opportunity to speak to physicians in a group setting. We used a nonexperimental quality improvement intervention design to identify baseline differences between participants who attended multiple classes and those who attended only 1 class. Participants who attended multiple classes, most of whom were Black men, achieved an average blood pressure reduction of 19.1/14.8 mm Hg. Although the classes were effective, current policies around health insurance reimbursement and federal quality reporting standards hamper the ability of health care providers to implement such patient education initiatives.

**Summary**

**What is already known about this topic?**

Interventions focused on patient education, skill-building, and self-management have been shown to be effective in combatting racial disparities in hypertension control.

**What is added by this report?**

This study presents results from the implementation of group-based hypertension self-management classes among patients of a federally qualified health center and identifies policy barriers to providing patient education resources.

**What are the implications for public health practice?**

Patients can make significant improvements to their blood pressure through continued reinforcement of culturally appropriate key concepts. Health care reimbursement and quality reporting systems should take steps to better encourage and support effective patient education interventions.

## Introduction

Hypertension is a significant risk factor for serious cardiovascular conditions such as stroke and heart failure (1). Reducing the burden of hypertension is a major public health goal in the US, especially in the populations in which it is most prevalent. Racial disparities in hypertension prevalence are extreme, with 42.4% of Black adults in the US diagnosed with the disorder compared with just 29.2% of White adults and 29.0% of Hispanic adults (2).

According to National Committee for Quality Assurance guidelines, controlled hypertension is a systolic blood pressure (BP) of <140 mm Hg and a diastolic BP of <90 mm Hg. Improving population hypertension control is challenging, with efforts further constrained by persistent racial disparities in socioeconomic factors and access to health care (3–5). After decades of structural racism in the form of social discrimination, economic marginalization, and resultant toxic stress, having uncontrolled hypertension is more common among racial minority groups, particularly among Black people (3–5). Nationally, hypertension is controlled among 67.3% of hypertensive White Federally Qualified Health Center (FQHC) patients and 67.1% of hypertensive Hispanic FQHC patients compared with only 57.0% of hypertensive Black FQHC patients (2). Additionally, uncontrolled hypertension is more common among men and people in the lowest income quintile (6).

These disparities provide cause to focus interventions on improving patient education, skill-building, and self-management among minority populations, particularly Black populations. Several strategies have been effective in lowering BP among high-risk populations. These include self-management education to give patients the skills to self-monitor their BP and improve modifiable risk factors such as diet and exercise (7,8), group-based education where patients can learn from peer experiences (8), and more effective medication management through regular contact with health care providers (9,10).

## Purpose and Objectives

The hypertension control strategies of self-management education, group-based education, and increased provider contact have been effective among Black men, underscoring the need for outreach and targeted interventions to reduce the hypertension disparity (7–10). Because our urban FQHC in the southeastern US sees more than 8,000 patients with hypertension per year, most (63%) of whom are Black, we were an ideal setting for outreach to this highest-risk population. Our objective was to describe attendees at our hypertension self-management intervention’s first-year classes, assessing class impact on participants’ BP and the success of the classes in encouraging repeat attendance.

We conducted the intervention in our urban FQHC, which in 2019 served 36,361 patients, 92.5% of whom were racial or ethnic minorities (11). More than half our patients were uninsured, 23.9% were on Medicaid, and 8.8% were on Medicare (11). Only 2% of patient households reported income at or above 200% of the federal poverty level (11). Among adult patients, 30.4% had a diagnosis of hypertension, and 59.3% of that number had controlled hypertension (11). Our center is located in a predominantly Black community and offers free transportation service for patients who live in the county to attend medical appointments.

## Intervention Approach

Group classes were held weekly on Tuesdays at 4 PM at the FQHC’s main site. The priority population for the intervention was Black men with severe hypertension (systolic BP >160 mm Hg or diastolic BP >100 mm Hg). These men were invited by telephone calls made by student volunteers; however, the class was open to all patients with hypertension, and most patients who attended learned of the classes through their primary care provider.

Classes were led by different clinicians each week. These included physicians, registered nurses, a nutritionist, and students in various health professions. Didactic content focused on 4 rotating topics: 1) defining hypertension, 2) the effects of hypertension, 3) how to eat for better heart health and lower BP, and 4) an “ask the experts” session. Each class discussed risk factors that could be modified to help control or lower BP. These lessons were given through live presentations, videos by organizations such as the American Medical Association (AMA) and the American Heart Association (AHA), and discussions centered around participant concerns. Participants attending for the first time were given an OMRON 3 Series BP monitor to use at home and were trained in BP self-monitoring in accordance with the joint recommendations of the AHA and the AMA (12). At each class, participants took 2 BP measurements 2 minutes apart, and the lower reading was recorded. Participants whose BP measured significantly above their personal goal were able to speak immediately to a family physician and adjust medications through the on-site pharmacy if necessary. A full description of the project implementation has been published elsewhere (13).

## Evaluation Methods

Our intervention used a nonexperimental quality improvement design to evaluate the program’s 28 classes. The primary outcome of interest was BP change among participants who attended multiple classes (multiple attenders), which could indicate an association between class attendance and BP reduction. To determine whether the classes were more engaging or more accessible to certain groups than others, a secondary analysis identified baseline differences between participants who attended multiple classes and those who attended only 1 class (single attenders).

Descriptive statistics were used to report baseline characteristics of study participants. Mean and standard deviation were used for continuous variables such as BP, and frequencies and percentages were used for categorical variables such as race/ethnicity. Inferential statistics were used to determine whether any baseline differences existed between participants who attended multiple classes and those who attended only 1. Paired *t* tests were used to compare means, and 2-sample *t* tests were used to compare proportions. These tests were conducted with Stata/SE version 16 (Stata Corp). The study was evaluated by the Duke University Institutional Review Board and deemed exempt as a quality improvement initiative.

## Results

Weekly hypertension classes were held at the FQHC from August 2019 through March 2020, at which point the COVID-19 pandemic forced the cancellation of in-person events. Over these 28 classes, 93 participants attended at least 1 session (Table 1). Sixty participants identified as Black, and 28 identified as Hispanic. Among all participants, the mean age was 57 (SD, 13); Of the 93 attendees, 38 Black men attended a single session (Table 2). Nineteen reported that they were already self-monitoring their BP before attendance, but most (58) were not self-monitoring, and 16 did not report. The mean BP for the group was 148/92 mm Hg (SD, 24.9 for systolic; 13.8 for diastolic).

**Table 1:** Baseline Characteristics of Attendees (N = 93) at Lincoln Community Health Center Hypertension Self-Management Classes (N = 28), August 2019 –March 2020

Characteristic	n (%)
**Age, y**	
<50	26 (28.0)
50–59	24 (25.8)
60–69	28 (30.1)
>70	15 (16.1)
**Sex**	
Female	41 (44.1)
Male	52 (55.9)
**Race/ethnicity^a^**	
Black	60 (64.5)
Hispanic	28 (30.1)
White	4 (4.3)
Unanswered	1 (1.1)
**Self-monitoring blood pressure prior^b^ to taking class(es)**	
Yes	19 (20.4)
No	58 (62.4)
Unanswered	16 (17.2)
**Insurer**	
Medicaid	11 (11.8)
Medicare	16 (17.2)
Private	15 (16.1)
None	46 (49.5)
Unknown	5 (5.4)

a Mean baseline systolic blood pressure of participants was 147.5 mm Hg (SD 24.9); mean baseline diastolic blood pressure was 91.6 mm Hg (SD 13.8).

b Patients were asked to self-identify, with the potential to leave the question blank.

**Table 2:** Baseline Characteristics of Attendees (N = 93) at Lincoln Community Health Center Hypertension Self-Management Classes (N = 28), Based on Attendance at Single or Multiple Classes, August 2019–March 2020^a^

Characteristic	Attended Single Class, n (%) (n = 66)	Attended Multiple Classes (n = 27)	*P* Value^b^
**Median classes attended**	1	3	–
**Mean age, y**	53.8	63.7	< .001
**Sex**			
Female	31 (46.9)	10 (37.0)	–
Male	35 (53.0)	17 (63.0)	–
**Race/ethnicity^b^**			
Black	37 (56.1)	23 (85.2)	.008
Hispanic	25 (37.8)	3 (11.1)	.01
White	3 (4.5)	1 (3.7)	–
Unanswered	1 (1.5)	0	–
**Self-monitoring blood pressure prior^c^ to taking class(es)**			
Yes	8 (12.1)	11 (40.7)	.002
No	44 (66.7)	14 (51.9)	< .001
Unanswered	14 (21.2)	2 (7.4)	–
**Insurer**			
Medicaid	9 (13.6)	2 (7.4)	–
Medicare	4 (6.1)	12 (44.4)	< .001
Private	10 (15.2)	5 (18.5)	–
None	38 (57.6)	8 (29.6)	.01
Unknown	5 (7.5)	0	–

Abbreviation: —, Not applicable.

a Mean baseline blood pressure of single-class attendees was 145.0 mm Hg (SD, 25.0) systolic and 90.2 mm Hg (SD, 13.0) diastolic. For attendees at multiple classes, baseline blood pressure was 155.8 mm Hg (SD, 25.1) systolic, and 95.0 mm Hg diastolic (SD, 13.3).

b Paired *t* tests determined significance for differences in mean (SD) between first class and final class. Two-sample *t* tests determined significance for differences in proportion between first class and final class. Values greater than P = .25 are not reported.

c Patients were asked to self-identify with the option of leaving the question blank.

Of the 93 participants, 27 attended multiple classes, 16 of whom were Black men. The average baseline BP of multiple attenders was 153.8/94.8 mm Hg, which was reduced to an average BP of 134.7/80.8 mm Hg by the end of class participation. This change represented an average reduction of 19.1 mm Hg in systolic BP (*P* = .004) and 14.8 mm Hg in diastolic BP (*P* = .002) over the course of their participation. Of the 27 people who attended the class multiple times, 24 achieved a reduction in systolic and/or diastolic BP.

Notable differences were seen in baseline measurements between those who attended multiple classes and those who attended a single class. The mean age of multiple attenders was 10 years older than that of single attenders (*P* < .001). Multiple attenders were mostly Black (23 of 27), and single attenders were more evenly Black and Hispanic people (37 of 66 and 25 of 66, respectively). Multiple attenders also had higher BP at baseline, with an average of 155.8/95.0 mm Hg compared with 145.0/90.2 mm Hg among single attenders, though this difference was not significant for either systolic (*P* = .06) or diastolic (*P* = .11) BP.

Black participants were significantly more likely to attend multiple classes than Hispanic participants (*P* = .004). Of 60 Black participants, 23 attended multiple classes, whereas only 3 of 27 Hispanic participants attended multiple classes. Participants who reported self-monitoring their BP before class attendance were significantly more likely to attend multiple classes than those who were not previously self-monitoring (*P* = .01).

## Implications for Public Health

Our group-based hypertension self-management classes were effective in reducing blood pressure among our priority population of Black men and were successful in encouraging repeat attendance. Because the risk of stroke has been shown to halve for every 20 mm Hg reduction in systolic BP, the average 19.1/14.8 mm Hg drop that multiple attenders achieved during the course of this study was a vital outcome (14). Almost all multiple attenders achieved some reduction in their BP. That success demonstrates the effectiveness of the course content for that group, which was predominantly Black, older, and more severely hypertensive than other attendee groups.

Participants aged ≤65 were less likely to attend multiple classes. This may have been due in part to work or childcare responsibilities. To mitigate this, classes could be held at multiple times of the day, other days of the week, or virtually to be more accessible to working patients.

We identified substantial policy barriers to sustainably implementing educational initiatives such as ours. First, current insurance reimbursement policies offer no incentives for clinicians to offer education classes. Private insurers still largely operate on a fee-for-service model that offers no support for educational interventions. Medicare reimburses FQHCs by patient visit by using the FQHC Prospective Payment System, which is tied to the cost of care and is updated annually. Medicaid reimbursement varies state-by-state but historically has not been tied to the quality of care provided. The revenue stream from which funds for education activities would likely come is Federal Section 330 funding, which typically goes toward covering care for the uninsured and for nonclinical services such as transportation. However, these funds are probably insufficient to cover new chronic disease management programs, especially during an economic period of declining health insurance coverage.

Another barrier is due to the failure of federal quality reporting standards to include the entire clinical picture. Universal Data System standards do not accept patient-reported measures of blood pressure, which are an efficient and safe measure of hypertension control, especially given the effects of the COVID-19 pandemic, which may prevent in-person clinical measurement. Patient-reported BP is particularly important for people with low incomes, especially those without insurance, who are unlikely to be able to afford frequent visits with their health care provider (15). Allowing for patient-reported BP measures using a cuff validated by a clinician would enable providers to be appropriately rewarded for the health benefits of group classes and similar interventions.

As a nonexperimental quality improvement intervention, our study has several limitations. We did see anecdotal evidence that multiple attenders implemented much of the knowledge they gained in the classes (13). However, we did not collect structured data on participants’ actions outside of the classes. As such, we cannot conclude whether BP reductions were due to medication adjustments, behavior changes, or factors entirely external to the classes. We did not analyze participants’ health factors aside from hypertension; doing so may have shed further light on differences between multiple and single attenders. We did not collect BP measurements after patients stopped attending classes, so our analysis cannot make any conclusions about the sustainability of participants’ BP improvements. Additionally, our class was held during the workday, which likely presented a barrier to attendance for working patients. Finally, because attendance was voluntary, our sample represented people who were ready to learn and engage around the hypertension issue. Therefore, our convenience sample is not generalizable to all hypertensive patients.

Further research is needed on how FQHCs can be proportionally rewarded for providing beneficial health programs that are not office visit–based. Insurers’ ongoing shift to value-based care will create an opportunity to focus resources on needed patient education activities. Attending multiple classes was associated with significant improvements in BP. Our findings support continued efforts by providers to reduce racial health disparities through patient education and self-management skill-building.
