# Erratum, Vol. 18, July 15, 2021 Release

**DOI:** 10.5888/pcd18.200628e

**Published:** 2021-09-02

In the article “Efficacy of Hypertension Self-Management Classes Among Patients at a Federally Qualified Health Center,” because of an author error, the name of a coauthor was misspelled in the author list and Acknowledgments. Author name Joan Chaplain, RN, has been corrected to Joan Chaplin, RN. The article was corrected on August 19, 2021, and can be found at https://www.cdc.gov/pcd/issues/2021/20_0628.htm. We regret any confusion or inconvenience this error may have caused.

